# Change in the perceived reproductive age window and delayed fertility in Europe

**DOI:** 10.1080/00324728.2023.2298678

**Published:** 2024-03-01

**Authors:** Ester Lazzari, Marie-Caroline Compans, Eva Beaujouan

**Affiliations:** University of Vienna (Wittgenstein Centre for Demography and Global Human Capital (IIASA, ÖAW, University of Vienna))

**Keywords:** fertility postponement, social age norms, maternal age, paternal age, Europe

## Abstract

While extensive literature documents the massive fertility delay of recent decades, knowledge about whether and how attitudes towards the timing of births have changed in Europe remains limited. Using data from two rounds of the European Social Survey, we investigate these changes and their association with macro-level fertility indicators in 21 countries. Between 2006–07 and 2018–19, societal consensus regarding the existence of optimal childbearing ages remained strong and became more in favour of later parenthood. Decomposition analyses show that these shifts were driven only partially by changes in population composition, supporting the idea that a general attitudinal change in favour of later childbearing is underway. We also find a trend towards gender convergence in upper age limits driven by the increasing social recognition of an age deadline for men’s childbearing. Although shifts in perceived reproductive age windows occurred during periods of birth postponement, they corresponded only loosely to country-level changes in fertility.

## Introduction

Since the 1980s, low-fertility countries have witnessed a decrease in average family size, in parallel with a rise in the mean age at which men and women start having children (Frejka and Sardon 2006). In line with these trends, numbers of births at advanced reproductive ages (i.e. ages 35+) have increased substantially (Prioux 2005). Late births per se are not a historically new phenomenon, as they were relatively common in the early 1950s, when they consisted mainly of higher-order births. What is exceptional about the ongoing trend in late fertility is that it concerns primarily first- and second-order births, due to the later start of family formation (Beaujouan 2020). Although these trends are common throughout Europe, they have taken place at different speeds and magnitudes across countries. For example, the share of births occurring at ages 35+ is relatively high in Southern Europe, but late childbearing remains less common in Central and Eastern European countries (Beaujouan and Sobotka 2017).

Age is often associated with notions about appropriate behaviours, and individuals within a society share common ways of thinking about the proper timing of events and the progression of roles over the life course (Neugarten and Hagestad 1976). There is evidence that age expectations are important in guiding various social behaviours, including the timing of family formation (Marini 1984; Blossfeld and Huinink 1991; Settersten and Hagestad 1996a, 1996b; Paksi and Szalma 2009; Billari et al. 2011; Van Bavel and Nitsche 2013; Kim and Cho 2021). These expectations are also subject to change over time and across contexts. Thus, studying their temporal and cross-national variations should be an integral part of understanding macro changes in fertility behaviours. Specifically, for periods when fertility has been massively delayed, the question of how perceived optimal ages for reproduction have changed gains relevance.

While the cited literature generally examines individuals’ perceptions regarding appropriate childbearing ages at one point in time, less attention has been devoted to temporal variation in perceptions. We take inspiration from the research by Billari et al. (2021), who documented the increase in average ideal ages at parenthood and upper age limits for childbearing using two rounds of the European Social Surveys (ESS) collected in 2006–07 and 2018–19. We extend this evidence by focusing on the change in social attitudes towards the timing of births across countries, comparing indicators of men’s and women’s optimal childbearing ages. We also introduce the concept of the ‘perceived reproductive window’ (i.e. the range of ages from the ideal age at first birth to the upper age limit for childbearing), and we link shifts in attitudes to observed changes in fertility behaviours.

Our first contribution is to examine changes over time in the ideal age for becoming a parent and the upper age limit (or age deadline) for childbearing in 21 European countries. We use three indicators that capture different aspects of change: the degree of consensus regarding the existence of ideal ages and upper age limits, and both the means and variabilities in the perceived optimal ages for having children. Given the increasing proportion of births occurring at advanced reproductive ages, we ask whether attitudes towards the timing of births have become less strict, that is, whether there is decreasing consensus about the existence of an optimal age for childbearing. Our second contribution is to explore potential sex differences in these patterns and investigate whether attitudes have shifted towards older ages for men and women alike. Our third contribution is to explore to what extent the shift in the perceived reproductive window is explained by compositional changes within a country (i.e. changes in population characteristics, such as education, employment, relationship status, childbearing timing, and religiosity) vs by a general diffusion of attitudes in favour of later fertility behaviours. Our fourth contribution is to investigate the associations between the temporal variations in ideal and observed mean ages at first birth and between perceived upper age limits for childbearing and late fertility.

## Background

### On the perception of optimal ages for childbearing

The life course is shaped by societal norms and shared beliefs regarding the appropriate ages for transitioning through different stages of life (Neugarten and Hagestad 1976). Descriptive social norms are beliefs about what people do or approve of, whereas social attitudes are internally motivated judgements (Park 2000; Cislaghi and Heise 2018). In the context of fertility timing, descriptive age norms may relate to a general public agreement regarding having children too early, to an ideal age at which it is considered preferable to become a parent, and to an age limit above which having children is socially discouraged (Settersten and Mayer 1997). Attitudes and norms are interconnected because, like norms, ‘preferences for the timing and sequencing of role changes are products of socialization, arising from internalization of the predominant behaviour patterns of significant others’ (Marini 1984, p. 234). However, confusion between social attitudes and social norms exists in the literature. In practice, it is difficult to separate attitudinal and normative components, as these are very dependent on each other. For instance, Park (2000) explained that social attitudes and social norms are closely related empirically, because they both involve making inferences about another person’s behaviour. The historical conflation of these two sociological concepts has resulted in the interchangeable use of the terms ‘attitudes’ and ‘norms’ in much of the literature on age structuring, without clear differentiation between the two (Liefbroer and Merz 2009; Paksi and Szalma 2009; Liefbroer and Billari 2010; Aassve et al. 2013; Kim and Cho 2021). Similarly, the ESS questions used in this study have been associated with capturing both social norms and social attitudes, although they elicit primarily individuals’ personal opinions about appropriate ages for life-course transitions, as indicated by the inclusion of phrases such as ‘in your opinion’. Hence, these questions reflect social attitudes rather than serving as a representation of broader societal constraints.

Previous studies using data from the third round of the ESS, collected in 2006–07, have documented the existence of perceived optimal ages for parenthood across Europe. Almost universally, individuals agree about the existence of a lower age limit for childbearing (Liefbroer and Merz 2009). Moreover, the mean perceived ideal age at first birth is considerably lower than the observed mean age at first birth, with an average gap of 3.4 years (Paksi and Szalma 2009). Most respondents also acknowledge the existence of social age deadlines for parenthood. These age limits tend to be higher for men (47.3 years) than for women (41.7 years). Furthermore, there is a greater consensus regarding the existence of age limits for motherhood than for fatherhood (Billari et al. 2011).

### The perceived reproductive window

In this study, we define perceived reproductive window as the ages at which people feel most comfortable about having a child in their social environment. This is the range of ages from the ideal age at first birth to the upper age limit for childbearing, rather than the ages between the lower and upper age limits (Liefbroer and Merz 2009). In low-fertility countries, where reproduction can be easily controlled and childbearing is predominantly a matter of personal preference, people typically postpone having children until at least their ideal age. As a result, the lower age limit for childbearing, which represents the minimum age for having children, is less relevant during periods of fertility delay. Conversely, the upper age limit is important, as it provides a threshold for individuals deferring their childbearing decisions and reflects perceived constraints when fertility is generally being delayed.

The answers to questions on ideal and upper age limits for childbearing may be influenced by several factors, including the perception of biological constraints. Women’s fecundity starts to decrease in their mid-20s and this decline accelerates in their mid- to late 30s (Habbema et al. 2015). Age-related infertility can also affect men, with the risk increasing especially after age 40 (La Rochebrochard et al. 2003; Sartorius and Nieschlag 2010). In addition to older parents facing more challenges in achieving a pregnancy, childbearing at older ages is associated with increased risks of pregnancy complications and poor infant outcomes (Kenny et al. 2013). Although most people tend to overestimate the age at which fecundity starts declining (both spontaneously and with infertility treatment), men and especially women are generally familiar with the notion that the ability to reproduce declines with age, as they estimate a lower likelihood of becoming pregnant at older ages (see Pedro et al. 2018, for a systematic literature review).

While biological considerations are in favour of an early start to family formation, young and teenage mothers are often stigmatized (Whitley and Kirmayer 2008; Yardley 2008). Having children too early can put the completion of education and achievement of financial stability at risk (Diaz and Fiel 2016), and it is socially expected that individuals will wait until they are established professionally and economically before having children (Blossfeld and Huinink 1991; Hoem et al. 2009). Another social pre-requisite for starting a family is formation of a stable relationship, and this is occurring at increasingly later ages in Europe (Vergauwen 2016), possibly exacerbated by prevailing ideologies of intensive parenting (Gregory and Milner 2011; Gauthier et al. 2021). At the same time, societal norms can discourage individuals from having children very late (Billari et al. 2011), as parents are expected to fulfil care responsibilities for several years after the birth of a new child.

### Are attitudes towards the timing of births becoming more flexible?

It is generally acknowledged that social norms and attitudes vary over time (Liefbroer and Billari 2010; Cislaghi and Heise 2018). According to sociological and demographic theories of individualization and modernization, such as the Second Demographic Transition (SDT), norms and attitudes in high-income countries have undergone rapid transformations since the 1970s (Van de Kaa 1987; Giddens 1992; Inglehart 2006; Lesthaeghe 2010). One underlying mechanism driving these changes is the influence of prevailing behavioural patterns on collectively held values (Marini 1984). As behaviours evolve, so do norms and attitudes towards family-related matters, due to the reciprocal relationship between attitudes/norms and actual family behaviours (Fishbein and Ajzen 1975; Ajzen 1991; Berrington et al. 2008). Consequently, the perceived optimal window for reproduction is dynamic and subject to variation over time. Given the ongoing trend of delayed fertility, it is likely that attitudes towards birth timing are also shifting towards later ages or becoming more flexible. In the context of individualization, the life course may be less structured by age-related norms imposed by social institutions, and social age attitudes may also relax.

Drawing on this literature, we put forward our first hypothesis, that attitudes towards the timing of births have become less strict (Hypothesis 1), as reflected by: (1) a lower proportion of respondents acknowledging the existence of optimal ages for childbearing; (2) a growing heterogeneity in the ages perceived as ideal and too late to have children; and (3) an overall increase in the perceived upper age limit for childbearing.

### Gender convergence in the perceived optimal ages for childbearing?

Our understanding of whether individuals hold more similar attitudes nowadays regarding the timing of childbearing for men and for women (i.e. gender convergence) is limited. An extensive literature on cultural change, focusing on gender norms in high-income countries, has shown that since the 1990s women’s and men’s social roles have become more equal (see e.g. Pampel 2011; Arpino et al. 2015; Goldscheider et al. 2015). These shifts have occurred alongside changes in women’s life courses, which have become more similar to those of men in terms of education and employment pathways (Lesnard et al. 2016). Also in the private sphere, although traditional cultural expectations about child-rearing persist (Langdridge et al. 2005; Gregory and Milner 2011; Goldscheider et al. 2015), there is evidence of a shift, with women spending less time on household chores and childcare than in the past (Pailhé et al. 2021). In line with the move towards a more gender-equal society and the declining differences between men’s and women’s late fertility patterns, we expect to find gender convergence in the perceived optimal ages for childbearing (Hypothesis 2).

### Compositional effects and attitudinal diffusion processes

The sociological literature has emphasized that cultural change can occur through the diffusion of new attitudes and behaviours in the population at large (Casterline 2001). Individuals’ reproductive decisions may be influenced either directly, by the reproductive behaviours of others who can provide concrete examples of the costs and rewards of making similar choices, or indirectly at a distance. For instance, advertisements in the mass media might bring assisted reproductive technology (ART) to the attention of individuals and increase their awareness of the possibility of having a child later in life through infertility treatment, hence providing an indirect mechanism for diffusion (Hornik and Mcanany 2001). In this way, the information gained through direct and indirect interactions can contribute to the broad diffusion of innovative behaviours (Montgomery and Casterline 1996; Casterline 2001). This provides a theoretical argument for explaining the documented positive country-level association between perceived upper age limits for childbearing and ART use (Billari et al. 2011; Kim and Cho 2021).

Attitudes also vary depending on individual characteristics and situational factors (Marini 1984; Jasso and Opp 1997; Liefbroer and Billari 2010). For instance, religious people are more likely to conform to traditional family values (Philipov and Berghammer 2007), but the size of these groups tends to shrink as societies become more secularized (Van de Kaa 1987; Inglehart 2006; Lesthaeghe 2010). Previous research has also found that more highly educated respondents are more likely to perceive higher upper age limits for childbearing compared with their lower-educated counterparts (Kim and Cho 2021), suggesting that a country’s educational expansion can be an additional driver of the spread of individualistic values (Lesthaeghe 2010). Attitudes regarding the timing of childbearing may also be influenced by circumstantial factors that shape individuals’ perceptions regarding whether a birth is ‘on time’ or ‘off time’: for example, employment conditions and relationship stability (Liefbroer and Merz 2009; Aassve et al. 2013). In sum, perceived optimal birth timings may shift because of the different characteristics of the population at different points in time (Palloni 2001).

To elaborate on these two mechanisms, we expect changes in perceived optimal ages to be determined partly by an attitudinal *diffusion process* and partly by *compositional changes* in population characteristics (Hypothesis 3). We use decomposition methods to investigate to what extent each mechanism explains the shifts in ideal ages for becoming a parent and social age limits for childbearing. Previous literature has found that shifts in population composition and in behaviours can both play relevant roles in driving changes in fertility indicators. For instance, the decline in marriage explains most of the increase in childlessness in the United States (Hayford 2013), and changing educational composition is an important factor explaining variations in cohort fertility rates across low-fertility countries (Lazzari et al. 2021), but the rise in childlessness in Europe is unrelated to educational composition (Beaujouan et al. 2016). Hence, we do not formulate any specific hypothesis regarding which component is more relevant to explaining the changes.

### How is the change in the perceived reproductive window related to delayed fertility?

Changes in attitudes and fertility behaviours are interrelated (Kohler et al. 2002; Berrington et al. 2008). As detailed earlier, when more people adopt new behaviours (e.g. having children later in life), the perceived reproductive window is likely to shift due to the diffusion of these new behaviours through social interactions (Kohler et al. 2002). Conversely, as perceived optimal ages for childbearing relax, births may occur later and later. The Theory of Planned Behaviour (Ajzen 1991; Liefbroer and Billari 2010) argues that changes in social norms and attitudes can be seen as precursors of changing behaviours. According to this view, social contexts that are more favourable towards later births may pave the way for fertility postponement and less traditional fertility behaviours, whereas stricter social norms and attitudes regarding childbearing may prevent them.

From a dynamic perspective, the final part of this paper asks to what extent shifts in the perceived optimal ages for childbearing are related to the postponement of births to later ages. Following the previous findings, we expect the magnitude of the attitudinal change to be larger in countries where childbearing has been postponed more (Hypothesis 4). Due to the nature of the data used, we do not attempt to assess the direction of causality, but we seek to provide a picture of variations in attitudes towards the timing of childbearing and their associations with late fertility trends over time and across countries.

## Data

We conducted comparative analyses for 21 countries that participated in both Round 3 and Round 9 of the ESS, administered in 2006–07 and 2018–19, respectively (www.europeansocialsurvey.org). The ESS is a biennial cross-sectional survey that captures attitudes and behaviours representative of the population aged 15 and over in each participating country. Each survey round consists of a core module that remains consistent across waves and rotating modules that periodically change. For this paper, we focus on the rotating module titled ‘The timing of life: the organization of the life course in Europe’, which includes some survey items specifically designed to ascertain childbearing age norms. About half of the respondents in each country are randomly assigned questions about women, while the other half are asked questions about men, using a ‘split ballot’ design. We use these two data points to evaluate changes in attitudes over time. Although a longer time span would be ideal for assessing trends, so far the ESS has only administered this module twice. Data on the mean age at first birth and age-specific fertility rates for women aged 15–49 by country are drawn from the Eurostat (2023) database. Given the absence of data on men’s fertility by age and parity over the period analysed, we focus on actual fertility only for women.

## Measures

By design, the survey captures respondents’ perceptions of the ages at which key family life events should or should not occur. Two questions serve as our main dependent variables:

- *In your opinion, what is the ideal age for a girl or woman [or a boy or man] to become a mother [or father]?*
- *After what age would you say a woman [or man] is generally too old to consider having any more children?*

Possible answers include: ‘No ideal age’, ‘Never too old’, a specific age (in integer numbers with no decimal points), and ‘Don’t know’. Refusals to answer the question are also recorded. While stating that there is ‘No ideal age’ or ‘Never too old’ may be a sign of flexibility in normative views, ‘Don’t know’ responses are more likely to reflect an absence of opinion on the issue.

Two aspects of the data collection process are worthy of attention. First, when respondents provide an age range as an answer, interviewers are instructed to ask for a specific age within that range. Since providing an age range can reflect more flexible attitudes regarding the timing of childbearing, there may be less strictness in attitudes than the data suggest. Second, the instructions given to interviewers on how to handle unanswered questions may vary across countries. While some survey organizations may instruct interviewers to use multiple probes when respondents leave questions unanswered, others may advise them to accept any answer provided (Koch and Blohm 2009). We trust that these variations do not significantly alter the distribution of numerical responses.

As in previous literature (Billari et al. 2011), our analysis excludes respondents who acknowledged extreme values, namely an ideal age at first birth below 12 years (*N* = 10) or an upper age limit below age 26 or above age 80 (0.9 per cent of the total sample). This exclusion aims to ensure that the outcomes of our study of change are not disproportionately influenced by the extreme values provided by a very small number of participants. Indicators of age norms at the macro level were constructed by aggregating individuals’ responses about men’s and women’s childbearing separately, by country and survey year.

While our analysis focuses on the ideal and upper age limits for childbearing, the ESS module also includes questions about early childbearing. Overall, there was a large consensus regarding the existence of a lower age limit for childbearing in both 2006–07 and 2018–19. On average, 80 per cent of respondents perceived a lower age limit of 20 or younger for entry into motherhood, while 60 per cent of respondents indicated a similar age threshold for entry into fatherhood (see Table A1, supplementary material).

Table 1 provides sample sizes for each analysed country. Building on the earlier discussion on social norms and attitudes, we interpret the aggregated responses to the two survey questions as a measure of the dominant preference within a country’s population regarding the timing of births.

## Methods

We use several indicators to investigate attitudinal changes regarding the timing of childbearing. First, the proportions of respondents acknowledging the existence of an ideal age for the start of family formation and an upper age limit for having children should mirror the extent to which members of a society share an attitude. Hence, an increase in the share of people not acknowledging an optimal age for childbearing (answering ‘No ideal age’ or ‘Never too old’ or simply not answering) may reflect a decrease in consensus. Second, a weakening of expectations regarding the optimal ages for childbearing can be found in an increased heterogeneity in the answers given.

In the first part of our analysis, we document variations in the strictness of attitudes towards the timing of births, measured by change either in the consensus towards the existence of optimal ages for childbearing or in the means and variabilities in the optimal ages for having children. We also construct a new indicator, the perceived reproductive window, corresponding to the ages between the mean ideal age for having a first child and the social upper age limit for childbearing.

In the second part of our analysis, we explore the extent to which the shift towards a preference for later childbearing has been driven by changes in population composition or diffusion of new attitudes by conducting a Blinder–Oaxaca decomposition (Blinder 1973; Oaxaca 1973) for the two optimal ages under study. Results from *t*-tests on the differences between the two survey rounds in mean perceived ideal ages and upper age limits for childbearing show the gaps to be statistically significant for most analysed countries (see Table A2, supplementary material). This indicates that we can meaningfully decompose changes in age norms over time.

Based on Ordinary Least Squares regressions, the Blinder–Oaxaca model allows us to decompose the shift in age norms between the two survey rounds into two components: (1) one part due to shifts in variable means, that is, compositional changes in domestic populations (explained component); and (2) a residual part due to shifts in variable coefficients, that is, behavioural changes across groups (unexplained component). This second part, unexplained by variations in observable population characteristics, reflects the diffusion in society at large of attitudes in favour of later childbearing. Originally used to investigate pay gaps and labour market discrimination based on race and sex, the Blinder–Oaxaca decomposition model has gained popularity in cross-national demographic research as a tool for distinguishing the extent to which macro-level social transformations are driven by compositional or behavioural factors (Yu 2015; Hook and Paek 2020; Pailhé et al. 2021).

Assuming that attitudes towards the timing of births, *A*, can be explained by a vector of variables, ***X***, we start by estimating separate linear regression models at times *t* and *t* + 1, as follows:.

(1) $$A_{t}=\alpha_{t}+\beta_{t}{\text{X}}_{t}+\varepsilon$$

and

(2) $$A_{t+1}=\alpha_{t+1}+\beta_{t+1}{\text{X}}_{t+1}+\varepsilon ,$$

where *α* is the intercept and *ε* is the error term. The mean difference in predicted outcomes between time *t* and *t* + 1 is then decomposed as follows:

(3) $$\begin{array}{c} {\bar{A}}_{t+1}-{\bar{A}}_{t}=\left(\alpha_{t+1}-\alpha_{t}\right)+\left(\beta_{t+1}-\beta_{t}\right){\bar{\text{X}}}_{t+1}\\ +\beta_{t+1}\left({\bar{\text{X}}}_{t+1}-{\bar{\text{X}}}_{t}\right) \end{array}$$

The first component is attributed to basic differences, the second refers to differences in coefficients (unexplained component), and the third part captures the portion of the difference in the means that is attributable to compositional changes (explained component). The underlying assumptions of the Blinder–Oaxaca decomposition model is that the explained and unexplained components have additive (independent) effects on attitudes, and differences in outcomes between the two groups are due only to differences in the characteristics included in the analysis. Hence, for the unexplained part to reflect the effect of behavioural changes correctly, all individual characteristics influencing attitudes towards the timing of births should be included in the model. To assess the statistical adequacy of the Blinder–Oaxaca decomposition, country-specific regression coefficients were examined and the assumption of homoskedasticity was tested using the Breusch–Pagan test.

Consistent with the literature discussed earlier, we assess the importance of the following situational factors that potentially affect individuals’ attitudes towards the timing of births: education (measured in years of completed full-time education); economic situation (self-reported evaluation of how comfortable it is to live on current income, on a four-point scale where ‘1’ means ‘Living comfortably on present income’ and ‘4’ means ‘Very difficult to live on present income’); relationship status (defined as a dummy variable, taking the value ‘1’ if the respondent is either married or cohabiting); an indicator of childbearing postponement (defined as a dummy variable taking the value ‘1’ if the respondent has reached age 30 without having a child and ‘0’ if the respondent has become a parent before age 30 or is still childless but has not reached this age at the time of the survey); and self-reported religiosity level (measured on a 10-point scale where ‘1’ means ‘Not religious at all’ and ‘10’ means ‘Very religious’). We also tested for the inclusion of other variables potentially relevant for explaining differences in attitudes among individuals, such as age and sex. However, these are omitted in the final model as their compositional contribution was negligible. We used the *oaxaca* command in Stata 17.0 (Jann 2008) to perform the decomposition analyses. The values of covariates across all countries are shown in Table 1.

In the last part of our analysis, we compare changes in perceived optimal ages with directly relevant indicators of *actual* fertility behaviours among women. Specifically, we examine: (1) the relationship between the change in the ideal age for becoming a mother and the observed mean age at first birth (MAB1) between 2006–07 and 2018–19; and (2) the parallel variations in the percentage contribution of births to women aged 40–49 to total fertility and the mean upper age upper age limit for childbearing between 2006–07 and 2018–19. The aim of this analysis is to explore whether correspondence exists between perceived optimal ages and fertility behaviours over time. In other words, are changes in attitudes accompanied by similar shifts in fertility behaviours?

## Results

### Consensus regarding the existence of optimal ages for childbearing remains strong

**Figure 1:**
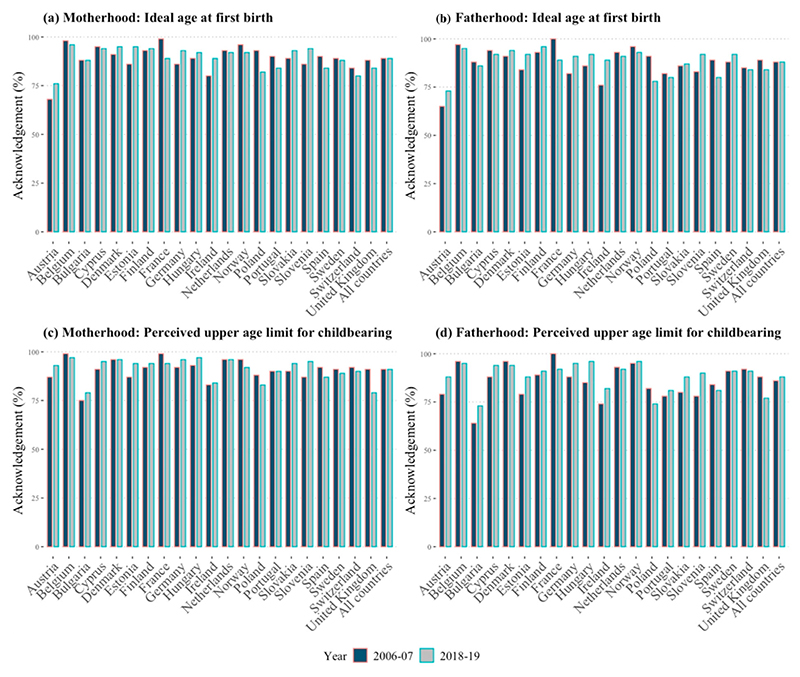
Acknowledgment of an ideal age at first birth and of an upper age limit for childbearing: European countries, 2006–07 and 2018–19 *Notes*: The figure shows the percentage of respondents who acknowledged an ideal age above 12 years and an upper age limit between 26 and 80. Data are weighted using analysis weights. Countries are presented in alphabetical order. For the numerical values, refer to Tables A3 and A4 (supplementary material). *Source*: Authors’ analysis of European Social Survey data (Rounds 3 and 9).

Most individuals across all countries recognized the existence of an ideal age for becoming a parent and an upper age limit for having children (Figure 1(a)–(d)). In most countries, less than one-fifth of respondents reported that there was no specific appropriate age for these childbearing events (Tables A3 and A4, supplementary material). We observe no important temporal changes in these proportions. Specifically, in all countries except Austria, the degree of consensus regarding the existence of an ideal age for entry into motherhood and fatherhood was consistently above 75 per cent in both 2006–07 and 2018–19, averaging 88–89 per cent (Figure 1 (a)–(b)). A strong consensus was also reported regarding the existence of upper age limits for childbearing, although larger for motherhood than for fatherhood (Figure 1(c)–(d)).

Moreover, we observe a convergence in the acknowledgment of an upper age limit for both men and women over time. This is the result primarily of a systematic increase in the share of respondents recognizing the existence of an age deadline for men’s childbearing but not women’s (Figure 1(c)–(d)). Indeed, all countries except for Denmark, France, the Netherlands, and Poland, show a decline in the gap between the proportion of respondents acknowledging the existence of an upper age limit for women’s childbearing but not for men’s. Five countries—Bulgaria, Hungary, Ireland, Slovakia, and Slovenia—which exhibit a large difference in the acknowledgement of age limits for men and for women in 2006–07 display a large reduction in this difference by 2018–19. The reduction ranges from three to seven percentage points, indicating a substantial convergence in views. Austria, Germany, and Portugal show more modest changes, with decreases of three percentage points. Notably, Finland exhibits no change, while Norway, Sweden, and Switzerland show a reversal in trend (an age limit was acknowledged more often for men than for women by 2018–19).

Measures capturing population heterogeneity in responses over time can also be used to evaluate changes in the consensus around a particular ideal age at first birth or upper age limit for childbearing. For instance, an increase in the interquartile range (IQR) for the ideal age at first birth and the age limit for childbearing will indicate a weaker consensus, while a decrease will suggest increasingly similar normative views. Across all countries, we observe a half-year increase in the IQR for the ideal age at first birth for women and a three-month increase for men (see Table A5, supplementary material). These patterns suggest a slight weakening of expectations regarding the appropriate age for starting a family. Responses on upper age limit for motherhood were more heterogeneous in the more recent ESS, but at least half of the respondents in most countries continued to provide an age limit between ages 40 and 45. For men, perceptions of an upper age limit are always more heterogeneous than for women. Nonetheless, their IQR declined in the later survey, with responses concentrated around ages 45–50. In other words, men’s perceived upper age limits have become less diverse and closer to those of women’s over time.

A final set of indicators suggests a weakening of expectations regarding upper age limits for childbearing, particularly for women. Smaller proportions of respondents in 2018–19 than in 2006–07 acknowledged the existence of an upper age limit for childbearing below age 40 for women and 45 for men in almost all countries (with decreases of 9.2 and 3.1 per cent, respectively, across all countries; Table A6, supplementary material). The decrease in these expectations was more substantial for women, another element that indicates a convergence in the perception of upper age limits for men and women over time.

### The shift towards later perceived optimal ages at birth

**Figure 2:**
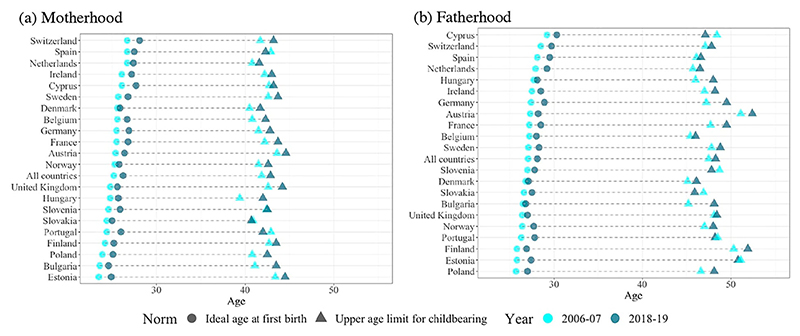
Change in the perceived reproductive window for motherhood and fatherhood between 2006–07 and 2018–19: European countries (a) Motherhood (b) Fatherhood *Notes*: Sample consists of respondents who acknowledged an ideal age above 12 years and an upper age limit between 26 and 80. Data are weighted using analysis weights. Countries are ordered by value of ideal age at first birth in 2006–07. For the numerical values refer to Table 2. *Source*: As for Figure 1.

Between 2006–07 and 2018–19, the ideal age at first birth for both men and women increased in all countries (Figure 2 and Table 2). The increases range from 0.3 to 1.6 years, with Cyprus, Switzerland, and Spain exhibiting the highest ideal ages for entry into both motherhood and fatherhood in 2018–19. Among countries with average ideal ages for motherhood under 25 years in 2006–07, only Bulgaria and Estonia still reported average ideal ages under age 25 in 2018–19, while ideal ages have increased above this threshold in Hungary, Poland, Portugal, Slovakia, Slovenia, Finland, and the United Kingdom (Figure 2 (a)). For men, ideal ages were already above 25 in 2006–07 and continued to increase, by one year on average by 2018–19 (Figure 2(b)).

This upward shift has been accompanied by a similar increase in perceived upper age limits for women’s childbearing (Figure 2(a)). Exceptions can be observed in Spain, Portugal, and Slovakia, where upper age limits have declined slightly. The mean maternal upper age limit in 2018–19 varied between 40.7 (in Slovakia) and 44.6 (in Austria). The mean paternal upper age limit does not exhibit a similar systematic increase; it has declined or remained mostly unchanged in 10 countries (Figure 2(b)). In 2018–19, the lowest mean paternal upper age limits were found in Slovakia (45.9) and Belgium (46.0 years), while the highest value was recorded in Austria (52.4 years), followed by Finland and Estonia.

**Figure 3:**
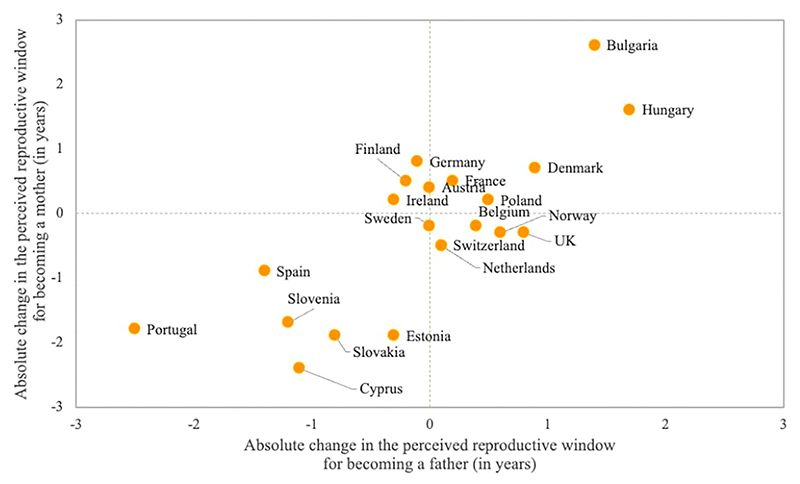
Association between the changes in the perceived reproductive windows for motherhood and father-hood between 2006–07 and 2018–19 *Notes*: Sample consists of respondents who acknowledged an ideal age above 12 years and an upper age limit between 26 and 80. Data are weighted using analysis weights. *Source*: As for Figure 1.

On average, men’s reproductive window was 3.7 and 3.5 years longer than women’s in Rounds 3 and 9 of the ESS, respectively. Changes in the length of the perceived reproductive window varied across countries, with notable increases in Bulgaria and Hungary and shrinking windows in Cyprus, Estonia, Slovakia, Slovenia, Spain, and Portugal. These changes have been influenced by shifts in both ideal ages at first birth and upper age limits for childbearing. In each country, changes in the reproductive window were generally of the same magnitude for men and women, as indicated by the good alignment along the diagonal in Figure 3.

### The diffusion of attitudes in favour of later births explains most of the shift in ideal and upper age limits for childbearing

We further investigate whether the general shift towards later perceived ages for parenthood between the two surveys was driven by a change in population characteristics within countries (composition effect) or whether individual attitudes favour later births more nowadays than in the past, irrespective of individuals’ characteristics (diffusion effect). The difference in means in social ages between the two ESS rounds is decomposed into two parts. The first reflects the mean increase in perceived optimal ages if the composition of the domestic population in 2006–07 had been the same as in 2018–19 in terms of five factors—namely, years of completed education, economic situation, relationship status, childbearing postponement, and religiosity level—that are likely to modify attitudes towards the timing of births (explained component). The second part reflects the hypothetical shift in 2006–07 when applying coefficients observed in 2018–19 (unexplained component); this will indicate whether new attitudes have broadly diffused within a country.

Among all countries analysed, the change over time in perceived optimal ages for childbearing has been driven mainly by a general shift in individuals’ attitudes towards later childbearing (unexplained effect; Table 3). This is particularly the case for men’s and women’s upper age limits. Table 3 shows important variations across countries. For instance, compositional changes explain only a tiny part of the shift towards later ideal ages for becoming a mother or a father in France, while this effect is more prominent in Bulgaria and Slovakia for mothers, and in Belgium, Hungary, Portugal, and Slovenia for fathers. In Switzerland, compositional factors would have driven a decrease in the perceived average ideal age for a first birth, but positive diffusion effects compensated for it, leading to an overall increase in perceived optimal ages for starting a family.

These models also enable us to disentangle *which* characteristics in a domestic population play a more significant role in explaining the observed changes in attitudes. In most countries, changes in the educational composition account for most of the (small) explained shift in perceptions towards a higher ideal age for becoming a mother or a father, whereas no main driver of the shift towards older upper age limits for childbearing is identified (for a visual representation of the results, see Figures A1–A4, supplementary material).

### The association between age norms and observed fertility behaviours

As attitudes regarding the ideal timing for childbearing are becoming more favourable towards later births by consensus, we finally focus on the aggregate relationship between changes in attitudes towards the timing of childbearing and changes in observed fertility indicators in the countries analysed. More precisely, we ask whether the magnitude of attitudinal change has been larger in countries where childbearing has been delayed more, with our focus on women.

**Figure 4:**
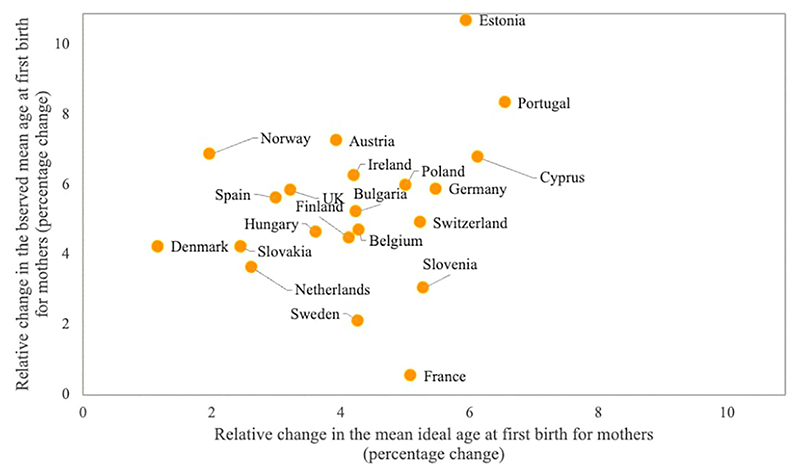
Association between the relative increases in the observed mean age at first birth and the mean ideal age at first birth for women between 2006–07 and 2018–19: European countries *Notes*: Sample consists of respondents who acknowledged an ideal age above 12 years and an upper age limit between 26 and 80. data are weighted using analysis weights. *Source*: For observed values: Eurostat (2023); Kreyenfeld et al. (2011) for Germany, 2006–07; Human Fertility Database (2023) for Denmark, 2006–07; Office for National Statistics (2023) for the UK, 2006–07. For ideal values: European Social Survey (Rounds 3 and 9).

At each time point, there was a strong and consistent positive association between mean perceived optimal ages at first birth and actual timing of first births at the country level (see Table A7, supplementary material). Indeed, countries with higher observed MAB1 were also countries in which respondents expressed higher mean ideal ages at first birth, with no outliers. This positive association is observed in both years, as indicated by correlation coefficients of 0.88 in 2006–07 and 0.83 in 2018–19. However, over time, changes in average observed and ideal ages at first birth are only weakly associated. As displayed in Figure 4, larger relative increases in mean ideal age at first birth are associated with higher relative increases in observed MAB1, but there are several outliers. For instance, Estonia displays a more pronounced upward shift in MAB1 than in the perceived optimal age for becoming a mother. In contrast, France, Sweden, and Slovenia experienced modest changes in observed MAB1, despite marked increases in the perceived ideal age at first birth.

In addition, a high share of respondents perceived an ideal age for becoming a mother that was lower than the observed MAB1, a discrepancy that increased over time (results for each country are reported in Table A7, last two columns). In 2006–07 between 48 and 81 per cent of individuals in each country stated that women should become mothers at an age below the national observed mean age at first birth, while these proportions increased to between 60 and 83 per cent in 2018–19. Overall, ideal ages at childbearing remained well below the observed mean age at first birth, with a gap of two and a half years in 2006–07 and three years in 2018–19 on average across all countries.

Shares of late births and perceived upper age limits are only loosely correlated cross-sectionally, and their increases over time do not display specific country patterns (Table 4). Specifically, we do not observe a cross-national correlation between the perceived upper age limit and the share of births occurring after age 40 in 2006–07, nor in 2018–19. In addition, there is no cross-national correlation in this increase. For instance, in Spain and Portugal, although contributions of late births to total fertility have expanded considerably, expectations regarding the most appropriate childbearing ages have become stricter over time (i.e. the ideal upper limit has decreased). By contrast, Cyprus and Estonia also exhibit high increases in late births, but perceived optimal ages for childbearing have shifted only moderately to higher ages. Other countries, such as Bulgaria and Hungary, where late fertility was still uncommon in the 2000s, have witnessed a rapid increase in the share of births occurring at older ages, accompanied by a large increase in upper age limits for childbearing.

## Discussion and conclusion

Using data from two rounds of a multi-country cross-sectional survey, this paper has explored changes in the perceived reproductive windows for motherhood and fatherhood in Europe and their relationship with observed fertility behaviours between 2006–07 and 2018–19. We moved beyond existing cross-sectional studies of perceived optimal ages for childbearing by focusing on the variation in age norms over time and by investigating whether correspondence existed between changing attitudes and childbearing behaviours at the country level.

Our first contribution was to explore whether societal expectations regarding the age at which men and women should have children have become more flexible, using multiple indicators capturing different aspects of change. From our analysis it emerged that social expectations regarding the appropriate age for childbearing are still relevant within the context of fertility postponement. Despite a rise in post-materialist and individualistic values (Inglehart 2006; Lesthaeghe 2010), consensus regarding their existence remained strong during the period of analysis. Not only did we not observe a decline in the proportion of respondents acknowledging the existence of age standards, we even documented a strengthening of perceived upper age limits for men’s childbearing over time. Heterogeneity in the ages perceived as ideal for entry into parenthood did not increase, with most responses falling within a narrow range of between three and five years, depending on the country. By contrast, IQRs for upper age limits for childbearing expanded slightly for women, from an average of 4.9 to 5.6, and narrowed for men, from an average of 8.8 to 7.3. Finally, between 2006–07 and 2018–19, the perceived reproductive window for motherhood and fatherhood shifted upwards, due to an increase in both the ideal age at first birth and the upper age limit for childbearing. The stable or increasing acknowledgment of age limits supports the idea that the shift in the perceived reproductive window towards older ages was not due to the population giving a specific numerical answer to the question becoming more selective over time.

Overall, Hypothesis 1 is only partially supported, as age expectations have weakened only in the sense that they have become more favourable to later parenthood and not because their existence is becoming less acknowledged than in the past. Although we were anticipating a decline in consensus regarding optimal childbearing ages, it is possible that the normative pressure to focus on self-actualization emphasized by the SDT has resulted in greater internalization of the age limits to childbearing, which may in turn have further reinforced the perceived importance of optimal childbearing ages.

Our second contribution was to investigate differences in the evolution of attitudes towards the timing of childbearing for men and women. Gender convergence occurred mostly through the reinforcement of social age attitudes regarding the timing of men’s childbearing. In 2018–19, respondents were more likely to acknowledge the existence of an upper age limit for men and within a smaller age range (between 45 and 50 years) but their likelihood of acknowledging the existence of an age limit for women was unchanged since 2006–07. This trend is in line with societal changes in gender roles and in the ongoing transformations in representations of fatherhood (Arpino et al. 2015; Goldscheider et al. 2015), which is perceived increasingly in relation to care activities and child-rearing and less through the idea that men have an unlimited biological lifespan in which to conceive. However, there was no gender convergence in terms of the ages perceived as ideal or too late to have children. Average gender gaps of two years for entry into parenthood and five years for the upper age limit for childbearing were maintained over time. This contrasts with trends in observed fertility behaviours, with women having displayed larger increases in fertility at older reproductive ages compared with men (Beaujouan 2020). As a result, Hypothesis 2 was also only partially supported.

Our third contribution was to examine whether the observed shifts in perceived optimal ages for childbearing were driven by changes in population composition. In partial support of Hypothesis 3, decomposition analyses revealed that the observed shifts were driven only marginally by composition effects, providing support to the idea that an attitudinal change more in favour of later childbearing is underway. This was the case both for ideal ages at first birth and for upper age limits for childbearing for men and women. The expansion in respondents’ educational attainment emerged as the most salient compositional factor modifying the perceived ideal timing of births. These patterns suggest that, in addition to individual and contextual reasons for having children at older ages, fertility delay has been facilitated by a transformation in subjective perceptions concerning the ideal ages for parenthood.

Our fourth and final contribution was to explore the relationship between changes in perceived optimal ages and shifts in actual fertility behaviours. Changes in perceived optimal ages have occurred during periods of fertility postponement, although not systematically hand in hand with observed fertility trends. As a result, Hypothesis 4 did not find support in our study. Indeed, while a consistent and strong positive cross-national correlation between ideal ages for becoming a parent and mean age at first birth was observed in each survey year, their variations over time were not closely linked. Upper age limits and the share of late births were in general not associated across countries, nor was their change. In some contexts, attitudes regarding age deadlines changed more than actual behaviours, whereas in other countries, births occurring over age 40 became more frequent even though having children at later ages was not more socially accepted. To establish a solid link between attitudes and behaviours, it may be necessary for the behaviour in question to be observed in a larger proportion of the population. While there has been an increase in the proportion of births occurring after the age of 40, this remains a relatively rare phenomenon, with most childbearing still occurring among individuals in their late 20s and early 30s. Therefore, it may be too early to observe a clear link between attitudes and behaviours.

Our results prompt a closer look at some specific cultural frameworks. For instance, Spain and Portugal exhibited marked upward trends in late births, although downward trends in perceived upper age limits for motherhood were seen over the period of analysis. In these Southern European countries, fertility has been delayed the most, hence the experience of infertility and of receiving unsuccessful ART treatments may be more common than in other countries and more likely to influence a downward revision of perceived upper age limits for reproduction. On the contrary, Hungary and Bulgaria experienced large absolute and relative increases in births over age 40, and their perceived reproductive window has also become more favourable to later births. These two Central European countries, where total fertility is low (between 1.5 and 1.6 children per woman in 2018–19), have eased access to ART since the early 2000s. In 2011, Bulgaria dropped an age-limit rule and now allows women of any age to undergo infertility treatment (Krastev and Mitev 2013), while Hungary increased public funding for fertility clinics in 2020 (Szalma and Djundeva 2020). Such events can influence public perceptions and contribute to an increasing acceptance of late births.

More generally, investigating institutional contexts can provide important information to improve our understanding of aggregated indicators of attitudes (Settersten and Mayer 1997). Differences in legal age limits for accessing ART and public insurance coverage have already been documented (Präg and Mills 2017), although their influence on perceptions is worth further analysis. Future research should investigate to what extent regulations are consistent with normative environments, whether a change in legislative frameworks can contribute to a shift in cultural views, and how the interaction between culture and institutional structures may explain cross-national differences in ongoing patterns of low fertility or recuperation. Qualitative frameworks could complement analyses of opinion surveys to provide a better understanding of to what extent and how perceived optimal ages for childbearing influence individual behaviours (Mynarska 2010; Perelli-Harris and Bernardi 2015).

Previous studies have assumed a causal relationship between changes in gender role attitudes and fertility trends, suggesting that new attitudes diffuse slowly and gradually influence behaviours (Feichtinger et al. 2003; Arpino et al. 2015). By contrast, our analysis did not address the direction of causality between childbearing attitudes and fertility trends but investigated concurrent changes in attitudes and behaviours. Previous research has also suggested that to be positively associated with fertility levels, attitudes towards a more gender-equal division of labour should not only be widespread but also shared by both men and women (Arpino et al. 2015). Our approach involved studying attitudes towards the timing of childbearing irrespective of the sex of the respondent. Further analysis could explore whether female or male respondents hold more traditional attitudes towards the proper timing of births and whether their attitudes are different in terms of women’s and men’s childbearing.

Although we controlled for variables typically associated with childbearing behaviours, we cannot rule out the possibility that other unobserved compositional factors may be influencing recent changes in attitudes. Still, the decomposition analysis does offer valuable insights into the extent to which typical predictors drive changes in attitudes. In general, while we can speculate on the causes of the unexplained portion of the gap in perceived optimal ages for childbearing and argue that this is due to diffusion mechanisms, the Blinder–Oaxaca decomposition model does not provide insights into which is the most plausible cause. In addition, the additive linearity assumption, which assumes that the different components of the decomposition analysis have additively separable effects on the outcome, may not hold in the presence of interaction effects. Finally, our study had limited capacity to establish a robust time trend due to data collection at only two points in time. While our findings indicate a shift in the perceived reproductive window in the context of delayed fertility, additional research is required to validate and contextualize this observation at different stages of the fertility transition.

The degree of consensus regarding optimal ages for childbearing and in particular upper age limits deserves closer examination because of its influence on fertility recuperation. Men and women may decide to give up on their childbearing plans, despite being still biologically able to have children, if the plans are perceived to be contrary to social expectations (Van Bavel and Nitsche 2013). From this perspective, the extent to which older reproductive ages are felt to be within the perceived reproductive window can act as either an obstacle or enabler for fertility recuperation at later ages. Hence, to understand the development of late fertility trends better, it is essential to continue examining the degree of consensus in populations regarding optimal ages for childbearing and to monitor the relationship between variations in attitudes and changes in observed fertility indicators. We found that shifts in attitudes regarding the ideal age of entry into motherhood were less pronounced (despite their lower original level) than actual shifts in MAB1. Moreover, first births were generally delayed to ages perceived as not ideal for having a first child, a phenomenon that became more pronounced with time as the trend towards childbearing postponement continued. This may signal a weakening of the regulatory power of age norms and their influence on fertility behaviours.

From a biological perspective, women’s reproductive lifespans are considerably shorter than men’s. For instance, a study of eight natural populations found that the maximum recorded age at last birth in women ranged between 45 and 58 years, while for men age at last reproduction ranged between 54 and 78 years (Vinicius et al. 2014). However, we found that the difference in the length of the perceived reproductive window between men and women was considerably shorter (less than four years) than the actual biological difference in their reproductive lifespans. While upper age limits for childbearing are increasing for both men and women along similar trajectories, the biological age constraint on parenthood is still stronger for women than for men, and ART does not appear to be able to compensate for this gap (Leridon and Shapiro 2017; Choi et al. 2023). Hence, new forms of gender inequalities in reproduction may arise in future as a result of a cultural land-scape increasingly more in favour of later childbearing for both sexes but which clashes with the uneven biological constraints that men and women face in becoming parents.

## Supplementary Material

Supplementary material

## Figures and Tables

**Table 1:** Sample sizes and descriptive statistics on covariates by country: European countries, 2006–07 and 2018–19

	2006–07						2018–19					
Country	Unweighted sample size	Years of full-time education completed (mean)	Economic situation (mean)	Relationship status (percentage not in a union)	Fertility postponement (percentage)	Religiosity level (mean)	Unweighted sample size	Years of full-time education completed (mean)	Economic situation (mean)	Relationship status (percentage not in a union)	Fertility postponement (percentage)	Religiosity level (mean)
Austria	2,402	12.5	1.8	54.5	25.4	5.1	2,491	12.6	1.8	54.3	31.1	4.8
Belgium	1,796	12.1	1.9	39.3	23.0	4.9	1,762	13.7	1.9	53.2	26.7	4.6
Bulgaria	1,391	11.2	3.1	39.9	15.0	4.3	2,320	11.7	1.6	47.8	21.1	4.2
Cyprus	992	11.3	2.1	33.5	19.4	7.0	801	12.1	2.3	38.3	28.0	6.9
Denmark	1,484	13.2	2.0	43.2	27.8	4.3	1,560	13.7	1.3	49.9	29.5	3.8
Estonia	1,516	12.2	2.3	56.2	17.3	3.6	1,897	13.4	2.1	60.0	17.7	3.3
Finland	1,894	12.4	1.9	49.2	26.9	5.3	1,750	14.0	1.8	51.7	30.1	4.9
France	1,936	12.5	1.9	47.8	25.6	3.7	2,002	13.1	2.0	55.8	30.1	4.7
Germany	2,907	13.2	2.0	46.2	29.0	3.9	2,347	14.3	1.7	45.3	34.1	4.2
Hungary	1,504	11.7	2.5	48.7	18.6	4.4	1,649	12.3	2.3	53.7	27.1	3.8
Ireland	1,743	12.7	1.7	51.4	40.3	5.4	2,207	14.5	1.8	48.7	44.7	4.9
Netherlands	1,884	13.2	1.7	47.7	36.2	4.9	1,665	14.1	1.5	46.3	35.5	3.9
Norway	1,744	13.4	1.5	47.7	23.6	3.8	1,403	14.0	1.5	54.1	33.6	3.2
Poland	1,713	11.5	2.3	44.6	13.3	6.5	1,554	12.7	2.1	43.2	23.2	6.1
Portugal	2,209	7.2	2.5	42.4	24.9	5.8	1,051	10.4	2.2	49.9	32.1	5.4
Slovakia	1,746	12.4	2.4	40.5	14.9	5.9	1,053	12.8	2.3	47.8	26.7	6.0
Slovenia	1,469	11.6	1.7	39.0	16.5	4.7	1,313	12.7	1.7	52.9	22.1	4.6
Spain	1,870	11.7	1.9	42.8	30.4	4.6	1,662	13.2	1.9	50.9	40.7	3.9
Sweden	1,917	12.6	1.5	55.5	26.7	3.5	1,531	13.7	1.4	50.5	31.5	3.1
Switzerland	1,837	13.4	1.6	45.4	39.2	5.5	1,533	11.3	1.6	48.8	41.5	4.7
UK	2,387	13.4	1.8	49.9	31.0	4.1	2,409	14.2	1.7	52.4	38.1	3.6

*Notes:* Data are weighted using analysis weights. The mean values for each variable are as follows: years of full-time education completed; economic situation (self-reported evaluation of income comfort on a four-point scale, where ‘1’ means ‘Living comfortably on present income’ and ‘4’ means ‘Very difficult to live on present income’); relationship status (dummy variable, ‘1’ if married or cohabiting); postponement of childbearing (dummy variable, ‘1’ if respondent has reached age 30 without a child); and religiosity level (measured on a 10-point scale, where ‘1’ means ‘Not religious at all’ and ‘10’ means ‘Very religious’).

*Source:* Authors’ analysis of European Social Survey data (Rounds 3 and 9).

**Table 2:** Ideal age at first birth and perceived upper age limit for childbearing in 2006–07 and 2018–19: European countries

	Ideal age at first birth				Perceived upper age limit for childbearing			
	2006–07		2018–19		2006–07		2018–19	
*Country*	*Women*	*Men*	*Women*	*Men*	*Women*	*Men*	*Women*	*Men*
Austria	25.4	27.3	26.4	28.2	43.6	51.1	44.6	52.4
Belgium	25.6	27.2	26.7	28.0	40.8	45.4	42.3	46.0
Bulgaria	23.6	26.5	24.6	26.8	41.1	45.2	43.5	48.1
Cyprus	26.1	29.2	27.7	30.3	42.7	48.4	43.2	47.1
Denmark	25.6	26.8	25.9	27.1	40.5	45.1	41.7	46.1
Estonia	23.5	25.8	24.9	27.4	43.4	51.1	44.5	50.8
Finland	24.2	25.8	25.2	26.9	42.7	50.3	43.5	51.9
France	25.5	27.2	26.8	28.5	42.2	47.7	43.7	49.5
Germany	25.5	27.4	26.9	28.9	41.5	47.2	42.8	49.5
Hungary	24.8	27.7	25.7	28.1	39.4	46.0	42.0	48.0
Ireland	26.1	27.5	27.2	28.5	42.2	47.0	43.0	48.2
Netherlands	26.7	27.9	27.4	29.2	40.8	45.7	41.6	46.5
Norway	25.3	26.4	25.8	27.7	41.5	47.0	42.6	48.0
Poland	23.9	25.7	25.1	27.0	40.8	46.6	42.5	48.1
Portugal	24.4	26.3	26.0	27.8	42.9	48.5	42.0	48.2
Slovakia	24.4	26.6	25.0	27.5	40.9	46.9	40.7	45.9
Slovenia	24.6	27.0	25.9	27.8	42.4	48.7	42.5	47.8
Spain	26.7	28.1	27.5	29.5	42.9	46.1	42.3	46.6
Sweden	25.7	27.1	26.8	28.3	42.6	47.8	43.7	48.8
Switzerland	26.7	28.5	28.1	29.7	41.7	47.1	43.2	47.8
UK	24.8	26.4	25.6	27.0	42.6	48.1	44.2	48.4
*All countries*	*25.2*	*27.1*	*26.2*	*28.1*	*41.9*	*47.5*	*42.9*	*483*

*Notes:* Sample consists of respondents who acknowledged an ideal age above 12 years and an upper age limit between 26 and 80. Data are weighted using analysis weights. Countries are presented in alphabetical order.

*Source:* As for Table 1.

**Table 3:** Estimated percentage contribution of explained and unexplained effects (from Blinder–Oaxaca decomposition models) to the change between 2006–07 and 2018–19 in the mean ideal age at first birth and upper age deadline for childbearing for women and men: European countries

	Ideal age at first birth				Perceived upper age deadline for childbearing			
	*Women*		*Men*		*Women*		*Men*	
*Country*	Explained	Unexplained	Explained	Unexplained	Explained	Unexplained	Explained	Unexplained
Austria	30*	70*	9	91*	−2	102*	2	98*
Belgium	34*	66*	46*	54*	23*	77*	91*	9
Bulgaria	46*	54*	111*	−11	5*	95*	4*	96*
Cyprus	16*	84*	13	87*	19	81	−17	117
Denmark	28*	72*	13*	87*	7*	93*	−1	101*
Estonia	18*	82*	19*	81*	3	97*	45	55
Finland	27*	73*	21*	79*	15*	85*	15	85
France	10*	90*	3	97*	16*	84*	18*	82*
Germany	16*	84*	16*	84*	11*	89*	13*	87*
Hungary	38*	62*	47*	53	4*	96*	−3	103*
Ireland	27*	73*	25*	75*	82*	18	39*	61*
Netherlands	39*	61*	19*	81*	6	94*	15	85*
Norway	35*	65*	18*	82*	14*	86*	25*	75*
Poland	27*	73*	19*	81*	13*	87*	19*	81*
Portugal	39*	61*	43*	57*	−19	119*	103	−3
Slovakia	55*	45	26*	74*	−13	113	2	98*
Slovenia	28*	72*	43*	57*	182*	−82	−41*	141*
Spain	25*	75*	20*	80*	−26*	126*	64*	36
Sweden	31*	69*	19*	81*	23*	77*	39*	61*
Switzerland	−11*	111*	−24*	124*	−22*	122*	−92*	192*
UK	33*	67*	35*	65*	15*	85*	71*	29

* *p* < 0.1.

*Notes:* Sample consists of respondents who acknowledged an ideal age above 12 years and an upper age limit between 26 and 80. Data are weighted using analysis weights.

*Source:* As for Table 1.

**Table 4:** Absolute and relative changes in the percentage contribution of fertility rates at age 40–49 to total fertility and in the perceived upper age limit for women’s childbearing between 2006–07 and 2018–19: European countries

	Late fertility indicators			Upper age limit indicators		
*Country*	Percentage contribution of fertility rates at age 40–49 to total fertility in 2006–07	Absolute change in indicator between 2006–07 and 2018–19 (percentage points)	Relative change between 2006–07 and 2018–19 (percentage change)	Upper age limit for female childbearing in 2006–07	Absolute change in indicator between 2006–07 and 2018–19 (years)	Relative change between 2006–07 and 2018–19 (percentage change)
Austria	2.4	1.8	72.2	43.6	1.0	2.3
Belgium	2.1	1.6	77.9	40.8	1.5	3.7
Bulgaria	0.9	1.6	171.0	41.1	2.4	5.8
Cyprus	2.5	2.8	111.8	42.7	0.5	1.2
Denmark	2.4	1.4	58.9	40.5	1.2	3.0
Estonia	2.3	2.6	114.1	43.4	1.1	2.5
Finland	3.1	1.4	46.6	42.7	0.8	1.9
France	2.9	1.4	49.0	42.2	1.5	3.6
Germany	2.6	1.8	69.0	41.5	1.3	3.1
Hungary	2.0	1.8	93.2	39.4	2.6	6.6
Ireland	4.8	1.7	35.9	42.2	0.8	1.9
Netherlands	2.3	1.3	57.9	40.8	0.8	2.0
Norway	2.4	1.4	57.1	41.5	1.1	2.7
Poland	2.0	0.7	36.3	40.8	1.7	4.2
Portugal	2.8	2.6	91.2	42.9	−0.9	−2.1
Slovakia	1.6	0.9	58.6	40.9	−0.2	−0.5
Slovenia	1.9	1.2	63.5	42.4	0.1	0.2
Spain	3.7	3.2	86.3	42.9	−0.6	−1.4
Sweden	3.1	1.2	39.6	42.6	1.1	2.6
Switzerland	3.2	2.0	63.2	41.7	1.5	3.6
UK	3.0	1.4	48.4	42.6	1.6	3.8
All countries	2.6			41.9		
Correlation coefficient		−0.36^a^	0.14^b^			

a Correlation between the absolute change in the percentage contribution of fertility rates at age 40–49 to total fertility and the absolute change in the upper age limit for female childbearing.

b Correlation between the relative change in the percentage contribution of fertility rates at age 40–49 to total fertility and the relative change in the upper age limit for female childbearing.

*Notes:* The absolute change refers to the difference in the indicator between 2006–07 and 2018–19. The relative change refers to the difference in the indicator as a percentage of the value of the indicator in 2006–07.

*Source:* For observed values: Eurostat (2023); Kreyenfeld et al. (2011) for Germany, 2006–07; Human Fertility Database (2023) for Denmark, 2006–07; Office for National Statistics (2023) for the UK, 2006–07. For ideal values: European Social Survey (Rounds 3 and 9).

## Data Availability

The data that support the findings of this study are openly available and can be downloaded from the ESS Data Portal after registration (www.europeansocialsurvey.org/) and from the Eurostat database (https://ec.europa.eu/eurostat/data/database).
