# Supplementary material for: Change in the perceived reproductive age window and delayed fertility in Europe
Source: Popul Stud (Camb). 2024 Mar 1;79(1):81–101. doi: 10.1080/00324728.2023.2298678 (PMC7616508; doi:10.1080/00324728.2023.2298678)
Supplement: Supplemental Material [file RPST_A_2298678_SM7819.pdf]

Supplemental Material

Table A1 Mean lower age limit for the childbearing: European countries, 2006–07 and 2018-19

	Motherhood				Fatherhood			
	2006-07		2018-19		2006-07		2018-19	
	Mean lower age limit	Share perceiving a lower age limit at or below age 20	Mean lower age limit	Share perceiving a lower age limit at or below age 20	Mean lower age limit	Share perceiving a lower age limit at or below age 20	Mean lower age limit	Share perceiving a lower age limit at or below age 20
Austria	18.4	86.1	18.8	83.8	19.9	71.9	20.6	66.8
Belgium	19.3	74.8	19.5	72.7	20.5	62.7	20.9	60
Bulgaria	19.2	81.1	18.8	85.5	21.3	56.3	20.9	67.3
Cyprus	19.5	79.8	20.5	67.1	21	58.3	22.4	44.6
Denmark	19.8	69.3	19.5	71.2	20.9	51.4	20.6	55.5
Estonia	18.8	84.9	18.9	81.8	20.4	67.1	20.8	63.8
Finland	19	83.8	18.9	83.4	20.3	69.5	20.6	64.9
France	19.4	76.2	19.4	77.4	21.1	59.5	20.8	61.2
Germany	19.1	80.8	19.2	75.1	20.6	61.9	21.2	57.2
Hungary	19.7	75.6	19.2	78.3	22.2	47.8	21.3	59.1
Ireland	20.7	57.9	19.9	65.3	21.9	45.5	21.4	53.7
Netherlands	19.5	80.3	19.7	69.7	20.8	59.3	21.4	50.7
Norway	18.9	86.9	18.6	84.6	19.9	74.8	20.1	68.5
Poland	18.7	85.2	18.3	89.4	20.2	66.1	20.3	65
Portugal	18.2	89.2	19.1	72.8	19.5	82.6	21.1	56.2
Slovakia	19	81.6	18.5	84.7	20.6	65.5	20.4	65.9
Slovenia	19.2	81.7	19.4	79.6	21.4	56.4	21.5	57.4
Spain	19.3	77.9	19.3	71.7	20.1	70.7	20.7	60.5
Sweden	19.4	79.9	19.7	76.7	20.9	62.5	21.1	76.7
Switzerland	19.3	79.6	19.4	74.4	20.9	61.4	20.7	58.9
United Kingdom	19	76.5	18.9	73.6	20.2	60.55	20.4	59
All countries	19.2	79.5	19.2	77.1	20.7	62.5	20.9	60.6

Notes: Data are weighted using analysis weights.
Source: European Social Survey data (Rounds 3 and 9).

Table A2 Two samples t-tests estimating the difference in mean values for the ideal age at first birth and upper age limit for childbearing: European countries, 2006–07 and 2018–19

	**Motherhood**		**Fatherhood**	
	Ideal age at first birth	Perceived upper age limit for childbearing	Ideal age at first birth	Perceived upper age limit for childbearing
Austria	***	***	***	**
Belgium	***	***	***	N.S.
Bulgaria	***	***	N.S.	***
Cyprus	***	N.S.	***	*
Denmark	N.S.	***	N.S.	*
Estonia	***	***	***	N.S.
Finland	***	*	***	N.S.
France	***	***	***	***
Germany	***	***	***	***
Hungary	***	***	N.S.	***
Ireland	***	*	***	**
Netherlands	***	*	***	*
Norway	**	***	***	*
Poland	***	***	***	***
Portugal	***	**	***	N.S.
Slovakia	*	N.S.	***	N.S.
Slovenia	***	N.S.	***	*
Spain	***	*	***	N.S.
Sweden	***	***	***	**
Switzerland	***	***	***	N.S.
United Kingdom	***	***	**	N.S.

Notes: N.S. = not statistically significant, *p-value<0.05, **p-value<0.01, ***p-value<0.001. Data are weighted using analysis weights.
Source: European Social Survey data (Rounds 3 and 9). SAMPLE: Respondents who acknowledged an ideal age above 12 years old, and an upper age deadline between 26 and 80 years old.

Table A3 Acknowledgment of an ideal age at first birth and of an upper age limit for childbearing: European countries, 2006–07

	**Motherhood**						**Fatherhood**					
	**Ideal age at first birth**			**Perceived upper age limit for childbearing**			**Ideal age at first birth**			**Perceived upper agelimit for childbearing**		
**Country**	Acknowledged	No ideal age	No answer	Acknowledged	Never too old	No answer	Acknowledged	No ideal age	No answer	Acknowledged	Never too old	No answer
Austria	68	23	9	87	3	10	65	24	11	79	9	12
Belgium	98	2	0	99	1	0	97	3	1	96	3	1
Bulgaria	88	3	8	75	8	17	88	2	10	64	16	19
Cyprus	95	4	1	91	3	6	94	4	2	88	6	6
Denmark	91	8	2	96	2	3	91	8	1	96	2	2
Estonia	86	10	3	87	6	7	84	11	4	79	13	7
Finland	93	6	1	92	5	4	93	6	1	89	7	3
France	99	0	1	99	0	1	100	0	0	100	0	0
Germany	86	9	5	92	2	6	82	13	4	88	7	5
Hungary	89	8	3	93	2	5	86	11	3	85	9	5
Ireland	80	16	4	83	5	12	76	20	4	74	13	13
Netherlands	93	4	2	96	1	3	93	5	1	93	4	3
Norway	96	3	1	96	1	3	96	2	1	95	3	2
Poland	93	3	4	88	3	9	91	4	5	82	9	9
Portugal	90	8	2	90	3	7	82	14	4	78	11	11
Slovakia	89	8	4	90	5	6	86	10	4	80	11	8
Slovenia	86	11	4	87	5	8	83	13	4	78	12	10
Spain	90	7	4	92	3	5	89	7	3	84	8	8
Sweden	89	8	3	91	4	5	88	9	3	91	4	5
Switzerland	84	14	2	92	3	5	85	14	1	92	4	4
United kingdom	88	9	3	91	3	6	89	7	4	88	8	4
All countries	**89**	**8**	**3**	**91**	**3**	**6**	**88**	**9**	**3**	**86**	**8**	**7**

Notes: Sample consists of respondents who acknowledged an ideal age above 12 years and an upper age limit between 26 and 80. The 'No answer' category includes two different types of item nonresponse: 'Don't know' (accounting for the largest part) and 'Refusal'. Data are weighted using analysis weights.
Source: European Social Survey data (Round 3).

Table A4 Acknowledgment of an ideal age at first birth and of an upper age limit for childbearing: European countries, 2018–19

	**Women**						**Men**					
	**Ideal age at first birth**			**Perceived upper age limit for childbearing**			**Ideal age at first birth**			**Perceived upper age limit for childbearing**		
**Country**	Acknowledged	No ideal	No answer	Acknowledged	Never too old	No answer	Acknowledged	No ideal	No answer	Acknowledged	Never too old	No answer
Austria	76	21	3	93	2	4	73	24	3	88	8	5
Belgium	96	3	1	97	2	1	95	4	1	95	3	2
Bulgaria	88	2	10	79	5	16	86	4	10	73	10	17
Cyprus	94	6	0	95	3	2	92	8	0	94	5	1
Denmark	95	4	2	96	1	3	94	4	3	94	3	4
Estonia	95	5	0	94	4	2	92	7	1	88	11	1
Finland	94	2	3	94	3	3	96	3	1	91	7	2
France	89	9	1	94	3	3	89	10	1	92	5	3
Germany	93	6	1	96	2	3	91	8	1	95	3	2
Hungary	92	6	2	97	1	2	92	6	2	96	1	3
Ireland	89	8	4	84	6	10	89	7	4	82	9	9
Netherlands	92	7	1	96	2	3	91	8	1	92	4	4
Norway	92	6	2	92	4	5	93	5	1	96	3	2
Poland	82	10	8	83	7	10	78	14	9	74	12	15
Portugal	84	14	2	90	7	3	80	18	2	81	14	5
Slovakia	93	5	2	94	3	3	87	10	3	88	8	5
Slovenia	94	4	1	95	2	3	92	7	1	90	7	3
Spain	84	12	5	87	6	7	80	14	6	81	11	8
Sweden	88	9	3	89	6	6	92	6	2	91	3	6
Switzerland	80	17	3	90	5	6	84	14	2	91	7	3
United Kingdom	84	8	9	79	6	15	84	6	9	77	6	17
All countries	**89**	**8**	**3**	**91**	**4**	**5**	**88**	**9**	**3**	**88**	**7**	**5**

Notes Sample consists of respondents who acknowledged an ideal age above 12 years and an upper age limit between 26 and 80. The "No answer" category includes two different types of item nonresponse: "Don't know" (accounting for the largest part) and "Refusal". Data are weighted using analysis weights.
Source: European Social Survey data (Round 9).

Table A5 Measures of dispersion in ideal ages at first birth and in upper age limits for childbearing: European countries, 2006-07 and 2018-19

	**Ideal age at first birth**												**Perceived upper age limit for childbearing**											
	Motherhood						Fatherhood						Motherhood						Fatherhood					
	2006-07			2018-19			2006-07			2018-19			2006-07			2018-19			2006-07			2018-19		
**Country**	Q1	Q3	IQR	Q1	Q3	IQR	Q1	Q3	IQR	Q1	Q3	IQR	Q1	Q3	IQR	Q1	Q3	IQR	Q1	Q3	IQR	Q1	Q3	IQR
Austria	25	28	3	25	28	3	25	30	5	25	30	5	40	45	5	40	50	10	45	58	13	50	60	10
Belgium	24	28	4	25	29	4	25	30	5	25	30	5	39	45	6	40	45	5	40	50	10	40	50	10
Bulgaria	22	25	3	23	26	3	25	28	3	25	30	5	40	45	5	40	45	5	40	50	10	45	50	5
Cyprus	25	28	3	25	30	5	27	30	3	28	31	3	40	45	5	40	45	5	45	50	5	45	50	5
Denmark	25	27	2	25	28	3	25	29	4	25	30	5	40	42	2	40	45	5	40	50	10	40	50	10
Estonia	21	25	4	23	26	3	25	28	3	25	30	5	40	45	5	40	49	9	45	55	10	45	55	10
Finland	22	25	3	23	27	4	24	28	4	25	30	5	40	45	5	40	45	5	45	55	10	45	55	10
France	24	28	4	25	30	5	25	30	5	25	30	5	40	45	5	40	45	5	42	50	8	45	51	6
Germany	23	27	4	25	30	5	25	30	5	26	30	4	40	45	5	40	45	5	40	50	10	45	52	7
Hungary	23	26	3	24	28	4	25	30	5	25	30	5	35	40	5	40	45	5	40	50	10	45	50	5
Ireland	25	29	4	25	30	5	25	30	5	25	30	5	40	45	5	40	45	5	40	50	10	45	50	5
Netherlands	25	30	5	25	30	5	25	30	5	27	30	3	40	45	5	40	45	5	40	50	10	40	50	10
Norway	24	27	3	25	28	3	25	29	4	25	30	5	40	45	5	40	45	5	40	50	10	45	50	5
Poland	22	25	3	24	26	2	25	27	2	25	30	5	40	45	5	40	45	5	40	50	10	45	50	5
Portugal	22	26	4	24	30	6	25	30	5	25	30	5	40	45	5	40	45	5	45	50	5	40	50	10
Slovakia	22	25	3	23	26	3	25	30	5	25	30	5	40	45	5	38	45	7	40	50	10	40	50	10
Slovenia	23	26	3	25	28	3	25	30	5	25	30	5	40	45	5	40	45	5	45	50	5	45	50	5
Spain	25	30	5	25	30	5	25	30	5	27	32	5	40	45	5	40	45	5	40	50	10	40	50	10
Sweden	25	28	3	25	30	5	25	30	5	25	30	5	40	45	5	40	45	5	45	50	5	45	50	5
Switzerland	25	30	5	25	30	5	26	30	4	28	32	4	40	45	5	40	45	5	42	50	8	44	50	6
United Kingdom	22	27	5	23	28	5	25	30	5	25	30	5	40	45	5	40	46	6	45	50	5	45	50	5
**All countries**	23.5	27.1	3.6	24.4	28.5	4.1	25.1	29.5	4.4	25.5	30.2	4.7	39.7	44.6	4.9	39.9	45.5	5.6	42.1	50.9	8.8	43.8	51.1	7.3

Notes: Sample consists of respondents who acknowledged an ideal age above 12 years and an upper age limit between 26 and 80. The interquartile range (IQR) is the difference between the 25th percentile (Q1) and 75th percentile (Q3). It provides information on the spread of the middle 50 percent. Data are weighted using analysis weights.
Source: European Social Survey data (Rounds 3 and 9).

Table A6 Share of respondents perceiving an upper age limit below age 40 for women and 45 years for men between 2006-07 and 2018-19, by country.

**Countries**	**Perceiving an age limit below age 40 for motherhood**			**Perceiving an age limit below age 45 for fatherhood**		
	2006–07	2018–19	change	2006–07	2018–19	change
Austria	37.1	32.2	-4.9	25.5	20.0	-5.5
Belgium	67.5	56.5	-11.0	60.2	57.5	-2.7
Bulgaria	58.2	41.9	-16.3	48.5	37.0	-11.5
Cyprus	51.8	45.1	-6.7	38.6	44.4	5.8
Denmark	68.6	54.7	-13.9	60.2	58.8	-1.4
Estonia	43.5	35.8	-7.7	25.9	27.1	1.2
Finland	49.7	42.3	-7.4	31.2	30.7	-0.5
France	55.5	40.3	-15.2	49.4	35.9	-13.5
Germany	59.8	48.4	-11.4	46.6	38.0	-8.6
Hungary	76.6	51.5	-25.1	50.9	44.6	-6.3
Ireland	51.3	44.5	-6.8	43.1	41.2	-1.9
Netherlands	65.8	57.6	-8.2	57.0	52.3	-4.7
Norway	56.7	48.2	-8.5	47.4	40.6	-6.8
Poland	66.3	44.5	-21.8	45.9	40.3	-5.6
Portugal	48.3	54.5	6.2	36.2	38.7	2.5
Slovakia	62.5	60.8	-1.7	41.7	48.1	6.4
Slovenia	53.0	50.4	-2.6	34.5	45.9	11.4
Spain	49.8	51.4	1.6	56.8	48.5	-8.3
Sweden	48.5	37.6	-10.9	44.7	38.7	-6
Switzerland	55.4	44.1	-11.3	48.8	41.2	-7.6
United Kingdom	49.9	40.1	-9.8	42.0	40.3	-1.7
All countries	56.0	46.8	-9.2	44.5	41.4	-3.1

Notes: Sample consists of respondents who acknowledged an ideal age above 12 years and an upper age limit between 26 and 80. Data are weighted using analysis weights.
Source: European Social Survey data (Rounds 3 and 9).

Figure A1 Estimated percentage contribution of the explanatory variables to the explained portion of the difference in mean ideal age at first birth for motherhood between 2006-07 and 2018-19: European countries

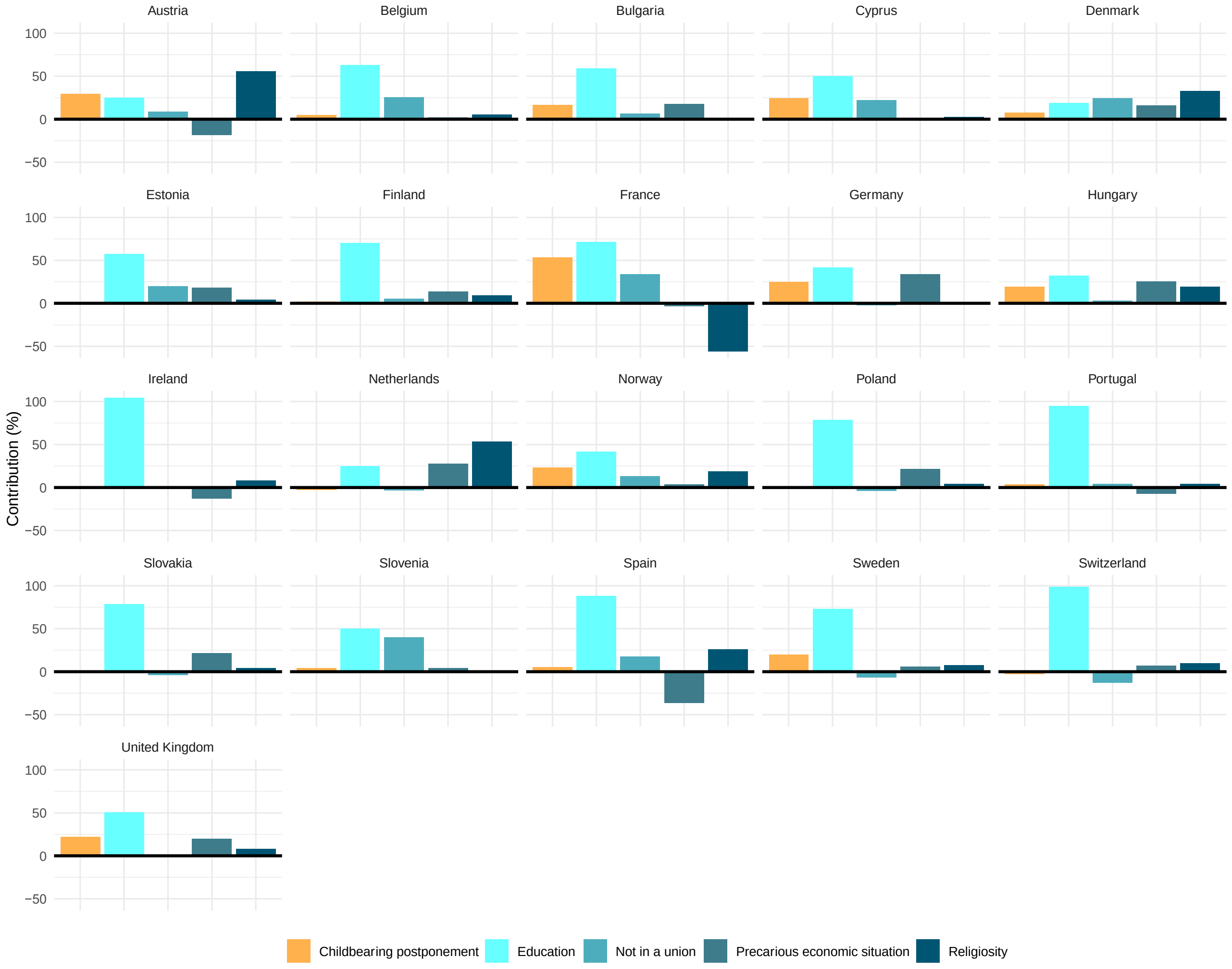

Notes: Sample consists of respondents who acknowledged an ideal age above 12 years and an upper age limit between 26 and 80. Data are weighted using analysis weights.
Source: European Social Survey data (Rounds 3 and 9).

Figure A2 Estimated percentage contribution of the explanatory variables to the explained portion of the difference in mean ideal age at first birth for fatherhood between 2006-07 and 2018-19: European countries

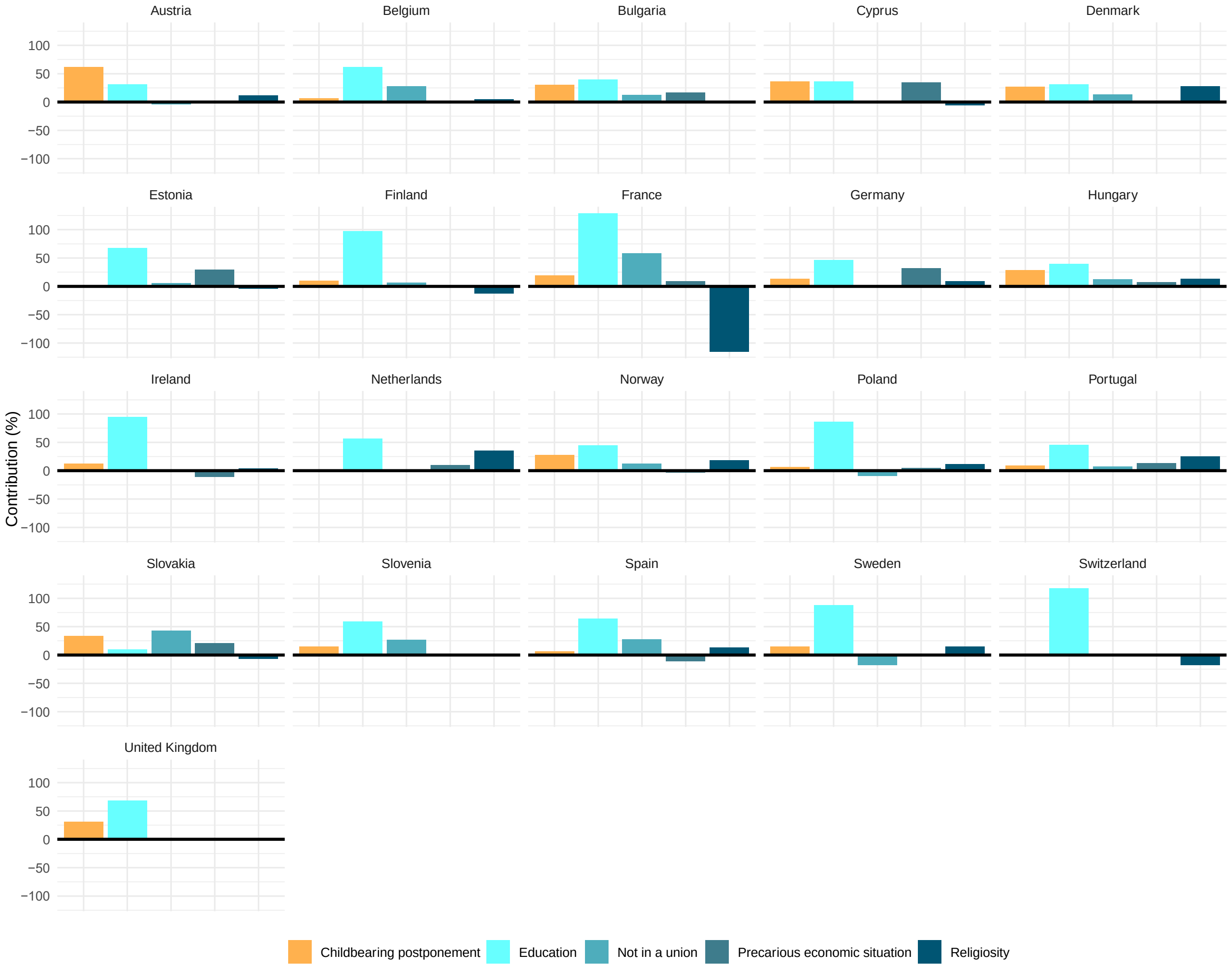

Notes: Sample consists of respondents who acknowledged an ideal age above 12 years and an upper age limit between 26 and 80. Data are weighted using analysis weights.
Source: European Social Survey data (Rounds 3 and 9).

Figure A3 Estimated percentage contribution of the explanatory variables to the explained portion of the difference in perceived upper age limit for the childbearing of women between 2006-07 and 2018-19: European countries

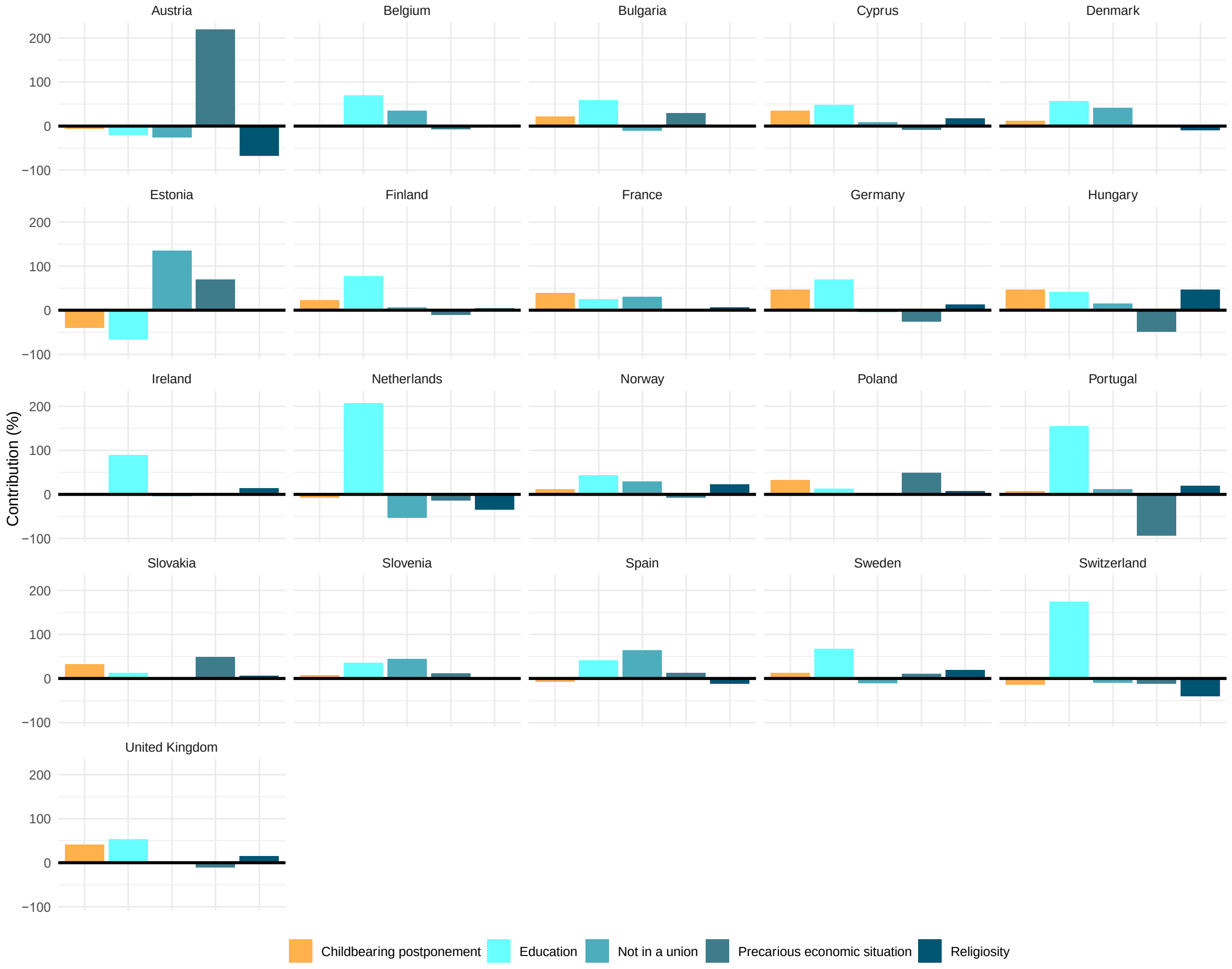

Notes: Sample consists of respondents who acknowledged an ideal age above 12 years and an upper age limit between 26 and 80. Data are weighted using analysis weights.
Source: European Social Survey data (Rounds 3 and 9).

Figure A4 Estimated percentage contribution of the explanatory variables to the explained portion of the difference in perceived upper age limit for the childbearing of men between 2006-07 and 2018-19: European countries

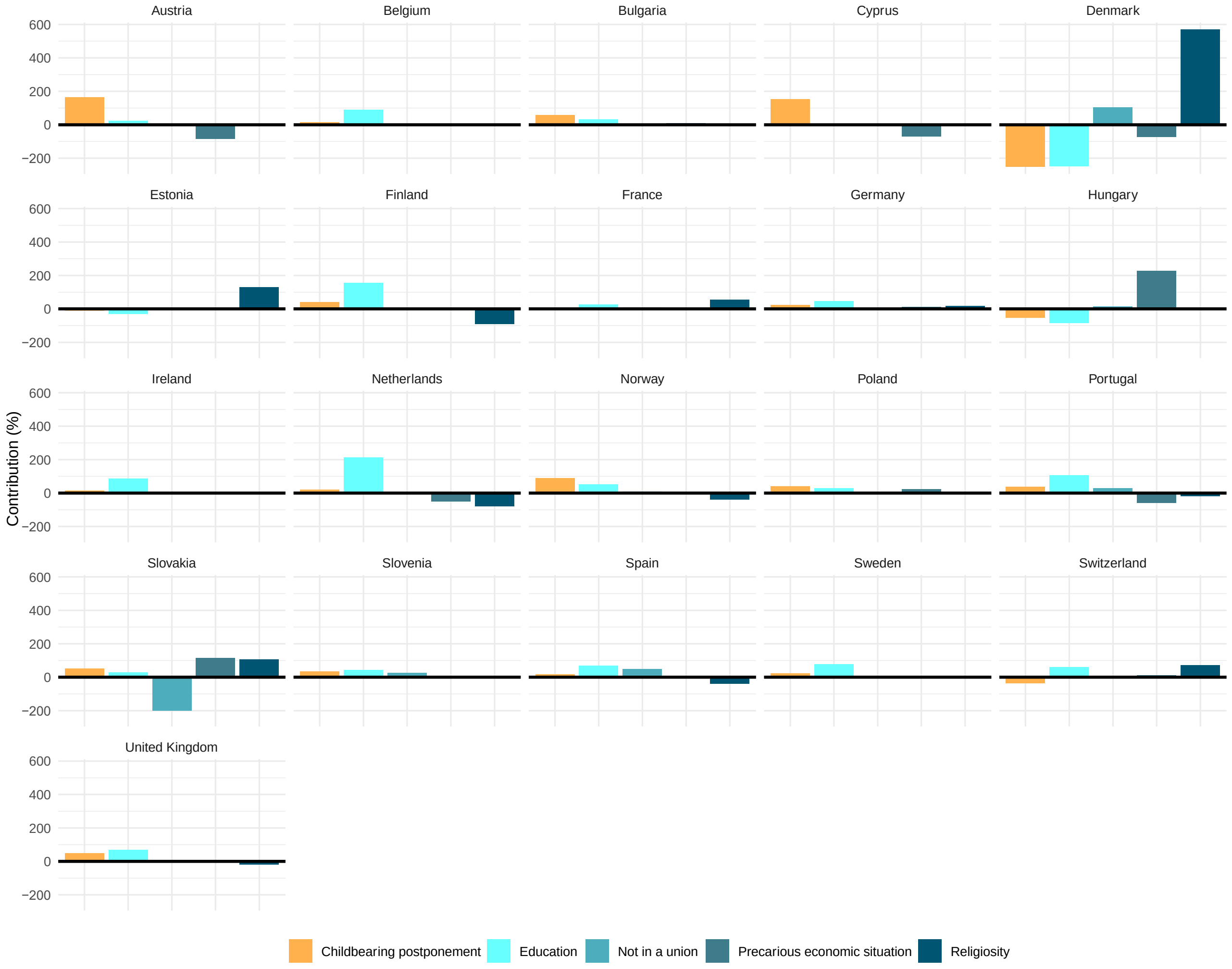

Notes: Sample consists of respondents who acknowledged an ideal age above 12 years and an upper age limit between 26 and 80. Data are weighted using analysis weights.
Source: European Social Survey data (Rounds 3 and 9).

Table A7: Observed and ideal mean age at first birth of women and share of respondents perceiving an ideal age at first birth lower than the one observed: European countries, 2006–07 and 2018–19

	**Ideal MAB1**		**Observed MAB1**		**Respondents with an ideal lower than the observed MAB1 (%)**	
**Country**	2006-07	2018-19	2006-07	2018-19	2006-07	2018-19
Austria	25.4	26.4	27.6	29.6	54.1	60
Belgium	25.6	26.7	27.8	29.1	72.2	71.8
Bulgaria	23.6	24.6	25	26.3	47.6	71.9
Cyprus	26.1	27.7	28	29.9	70.2	62.9
Denmark	25.6	25.9	28.4	29.6	69.7	81.9
Estonia	23.5	24.9	25.3	28	50.5	79
Finland	24.2	25.2	28.1	29.3	80	82.5
France	25.5	26.8	28.6	28.8	80.7	59.5
Germany	25.5	26.9	28.1	29.8	67.9	63.5
Hungary	24.8	25.7	27	28.3	70.8	70.5
Ireland	26.1	27.2	28.8	30.6	61.6	77.8
Netherlands	26.7	27.4	29	30.1	68.9	62.5
Norway	25.3	25.8	27.7	29.6	77.8	79.1
Poland	23.9	25.1	26	27.5	78.1	74.2
Portugal	24.4	26	27.6	29.9	76.5	61.9
Slovakia	24.4	25	26.1	27.2	69	72.7
Slovenia	24.6	25.9	28	28.9	74.8	77.1
Spain	26.7	27.5	29.4	31.1	64.5	76.2
Sweden	25.7	26.8	28.8	29.4	73.3	62.6
Switzerland	26.7	28.1	29.5	31	63.3	72.5
United Kingdom	24.8	25.6	27.4	29	64.2	70.1
All countries	25.2	26.2	27.7	29.2	68.4	71.0
Correlation coefficient	0.88[a]	0.83 [a]				

[a] between ideal MAB1 and observed MAB1

Notes: Sample consists of respondents who acknowledged an ideal age above 12 years and an upper age limit between 26 and 80. Data are weighted using analysis weights.
Source: For observed values: Eurostat (2023); Kreyenfeld et al. (2010) for Germany (2006–07); Human Fertility Database (2023) for Denmark (2006–07); Office for National Statistics (2023) for the United Kingdom (2006–07). For ideal values: European Social Survey (Rounds 3 and 9).
